# MRI- and report-based multimodal model with SHAP-based explanation for preoperative prediction of deep stromal invasion in early-stage cervical cancer

**DOI:** 10.1186/s13244-026-02311-7

**Published:** 2026-05-15

**Authors:** Raoying Xie, Yao Ai, Anqi Bao, Lihua Xie, Wenjing Wang, Kangwei Zhu, Jianping Wu, Yiyang Wu, Ruyan Xiong, Xiance Jin

**Affiliations:** 1 Department of Radiation and Medical Oncology, The First Affiliated Hospital of Wenzhou Medical University, Wenzhou, China; 2 Department of Radiotherapy and Chemotherapy, The second Affiliated Hospital of Wenzhou Medical University, Wenzhou, China; 3 School of Basic Medical Science, Wenzhou Medical University, Wenzhou, China

**Keywords:** Early-stage cervical cancer, Depth of stromal invasion, Radiomics, Natural language processing, Machine learning

## Abstract

**Objectives:**

Depth of stromal invasion (DSI) is a key prognostic factor significantly influencing treatment decisions in early-stage cervical cancer (ESCC). This study aims to develop an explainable multimodal data fusion model integrating MRI, radiology reports, and clinical variables for the preoperative assessment of DSI risk.

**Materials and methods:**

Radiomic features were extracted from preoperative sagittal T2-weighted imaging (T2WI). Bidirectional Encoder Representations from Transformers (BERT) features were derived from the corresponding radiology reports using natural language processing (NLP). Key BERT and radiomic features were selected using the least absolute shrinkage and selection operator (LASSO). Independent clinical risk factors were identified through univariate analysis, followed by multivariate logistic regression. Five machine learning algorithms were employed to construct the clinical (C), text (T), radiomic (R), and multimodal fusion models (T + R, T + R + C). The Shapley Additive exPlanation (SHAP) method interpreted the optimal fusion model.

**Results:**

Overall, 498 radiomic features (from T2WI) and 384 BERT-derived features (from reports) were extracted per patient. LASSO selected eleven BERT features and nine radiomic features; multivariate analysis identified two independent clinical risk factors. The text-radiomic-clinical fusion model (T + R + C) outperformed all other models, achieving AUCs of 0.912, 0.874, and 0.890 in the training, internal validation, and external validation cohorts, respectively. SHAP analysis revealed that eight BERT features, five radiomic features, and two clinical features ranked among the top 15 most influential predictors.

**Conclusion:**

The explainable multimodal model improves preoperative DSI risk evaluation in ESCC. However, given the small external cohort (*n* = 20), these results are preliminary and require further independent multi-center validation before clinical application.

**Critical relevance:**

The developed model significantly improves preoperative DSI prediction in ESCC through noninvasive imaging analysis, thereby refining risk assessment and guiding precision oncology interventions.

**Key Points:**

- DSI is a key prognostic factor significantly influencing treatment decisions in ESCC.
- The explainable multimodal fusion model was established.
- The accuracy of preoperative DSI risk evaluation was beneficial for clinical decision-making.

**Graphical Abstract:**

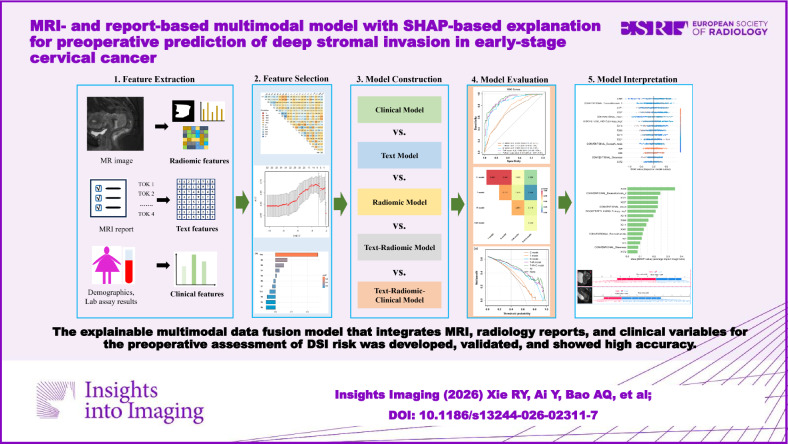

## Introduction

Cervical cancer (CC) is one of the most common cancers and the fourth leading cause of cancer-related death among women worldwide [1]. In recent years, the widespread implementation of CC screening programs has led to an increasing detection of early-stage cervical cancer (ESCC) (classified as International Federation of Gynecology and Obstetrics classification (FIGO) 2018 stages IB to IIA) [2]. Surgery or concurrent chemoradiotherapy is the primary treatment modality in the management of ESCC [3]. Current clinical decision-making relies heavily on risk stratification based on tumor size, depth of stromal invasion (DSI), and lymph-vascular space invasion (LVSI) [4, 5]. Specifically, limited radical hysterectomy is recommended for ESCC patients with a tumor size < 2 cm in diameter and no involvement of middle or one-third DSI, aiming to minimize treatment-associated complications [6]. However, patients with moderate or deep DSI often require adjuvant radiotherapy following radical hysterectomy, particularly when vascular involvement or other high-risk pathological features are present [7]. Notably, DSI is a critical prognostic factor influencing both surgical planning and postoperative management [8]. Therefore, preoperative identification of DSI is of great clinical value in the optimal management of ESCC.

Traditional medical imaging modalities, such as magnetic resonance imaging (MRI), ultrasound, and positron emission tomography/computed tomography (PET/CT), have been employed for preoperative DSI assessment in ESCC, yet their diagnostic accuracy (ACC) remains suboptimal [9–11]. Recently, Radiomics has been increasingly and widely applied in the diagnosis, prognostic evaluation of various cancers by extracting high-dimensional features from medical images [12, 13]. For instance, radiomics features extracted from MRI images demonstrated satisfactory diagnostic ACC in the preoperative prediction of DSI in comparison with traditional vision diagnosis and clinical factors [14–16]. Furthermore, multimodal approaches integrating radiomics with clinical factors have achieved improved sensitivity (SEN), specificity (SPE), and area under the curve (AUC) in comparison with radiomics-only models [17]. However, existing studies are predominantly limited by single-center designs and small sample sizes, constraining their generalizability.

Medical imaging reports (MIRs) are the interpretation of medical imaging information by radiologists, which contain rich disease information for supporting and confirming diagnosis in clinical practice [18]. However, the unstructured nature of MIRs hinders its wide application in the clinic, as manual extraction of data from free-text imaging reports is time-consuming [19, 20]. The advent of Transformer-based natural language processing (NLP) architectures has revolutionized automated information retrieval from unstructured texts, enabling efficient conversion of MIRs into structured formats for clinical and research applications [21–24]. The purpose of this study is to develop an explainable multimodal data fusion model that integrates MRI radiomics, NLP-processed MIRs data, and clinical variables for preoperative stratification of DSI risk in ESCC patients. By addressing the limitations of conventional imaging and single-modality analyses, our approach aims to establish a novel tool for rapid and accurate risk stratification, thereby assisting clinicians in making informed treatment decisions and ultimately improving patient outcomes.

## Materials and methods

### Patients

**Fig. 1:**
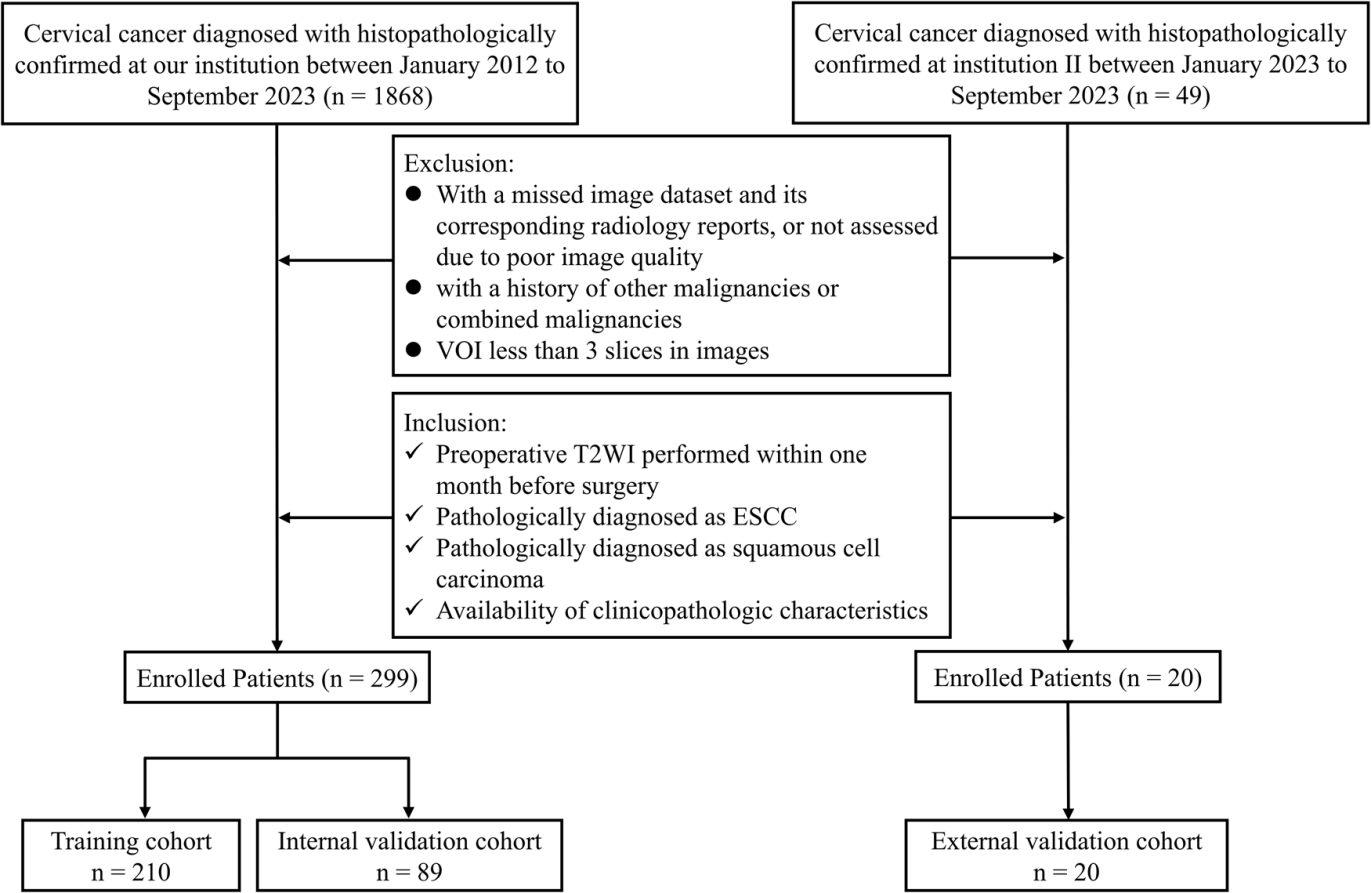
The flow chart of inclusion and exclusion criteria

This study enrolled 319 patients with ESCC from two centers between January 2012 and September 2023. Inclusion criteria were: (1) with preoperative sagittal T2-weighted imaging (T2WI) performed within one month; (2) pathological confirmation of ESCC; (3) pathologically diagnosed as squamous cell carcinoma; (4) availability of comprehensive clinicopathologic characteristics. Exclusion criteria were: (1) missing imaging datasets or radiology reports, or inadequate image quality for assessment; (2) history of other malignancies or concurrent malignancies; (3) volume of interest (VOI) spanning fewer than three slices. A flowchart of patient inclusion/exclusion is shown in Fig. 1. The methodological workflow is illustrated in Fig. S1. This retrospective study was approved by the Institutional Review Boards of all participating hospitals (ECRR no. KY2025-R058) with waiver of informed consent.

### Imaging and radiomic feature extraction

MRI was acquired using 3.0-T scanners (PHILIPS, ACHIEVA, Holland; GE, Signa EXCITE, America; GE, Signa HDxT, America) with phased-array body coils. Detailed acquisition parameters are presented in Supplementary Table S1. Images were initially resampled to 1 mm × 1 mm × 1 mm isotropic voxels. Intensity normalization was performed by subtracting the mean from each voxel and dividing by the standard deviation, scaling values to 0 - 600. Discretization was conducted using a fixed bin width of 3 for feature computation. Tumor volumes were manually delineated as regions of interest (ROIs) on each sagittal T2WI slice by a radiologist with over 7 years of experience in pelvic MRI, using LIFEx (v. 7.2.0; http://www.lifexsoft.org) [25]. Representative VOIs are displayed in Supplementary Fig. S2. To evaluate the interobserver reproducibility of the radiomic features, ROIs were radially expanded/eroded by 1.5 mm per slice in 40 randomly selected patients to simulate interobserver variability [26]. Features with an intraclass correlation coefficient (ICC) > 0.75 were selected for further analysis to ensure reproducibility. A total of 498 quantitative radiomic features were extracted from sagittal images using 9 different filtering modes via the LIFEx software in accordance with the Image Biomarker Standardization Initiative (IBSI) [27].

### MIRs feature extraction

MIRs were written in Chinese and used for feature extraction. These reports contained radiologist-provided descriptions of imaging findings and differential diagnoses. During pre-processing, raw report data were transformed into structured datasets to enable efficient feature extraction and analysis. First, to prevent potential label leakage and ensure the model did not rely on explicit diagnostic conclusions, a rigorous text-filtering protocol was implemented. Two senior radiologists (with 5 and 10 years of experience, respectively) identified a comprehensive list of keywords and phrases that explicitly or implicitly indicated DSI status (e.g., “full-thickness involvement,” “disruption of the fibrous stroma ring,” and descriptors of parametrial or vaginal invasion). These identified patterns were then systematically removed using a Python-based keyword-matching script. A detailed list of the excluded terms is provided in Supplementary Table S2. Next, radiologist names and timestamps were removed to preserve anonymity, and records with empty fields were excluded. Finally, each MRI report’s content was combined into a single line of text to facilitate downstream processing.

Bidirectional Encoder Representations from Transformers (BERT), a language representation model pretrained on large corpora, has gained significant attention for outperforming conventional NLP models by capturing long-range dependencies through an attention mechanism, which enables it to consider the entire input simultaneously rather than processing text in a strictly left-to-right or right-to-left manner [28]. Therefore, BERT is capable of learning contextual word embeddings and supporting feature extraction. In this study, Text2vec, a text-to-vector toolkit, was used for feature extraction from MR reports [29].

### Feature selection and clinical factors

The ICC was computed using MRI from 40 randomly selected patients to identify reliable radiomic features. Features exhibiting ICCs above 0.75 demonstrated satisfactory reproducibility and were chosen for model development. Subsequently, BERT and radiomic features were normalized through Z-score standardization to obtain a standard normal distribution of feature intensity [30].

A multi-step feature selection process was utilized to identify the key BERT and radiomic features within the training dataset. First, Spearman’s correlation analysis was applied to identify highly correlated features with |*p*| ≥ 0.8. Then, the Mann–Whitney *U*-test was used to select features with *p* < 0.05 as potentially informative features. Finally, the least absolute shrinkage and selection operator (LASSO) method with ten-fold cross-validation was applied to obtain the most significant features by compressing the coefficients of unrelated features to zero. Univariate and multivariate logistic regression analyses were applied to identify clinical factors associated with the risk of DSI in the training cohort (*p*-values < 0.05). All preprocessing steps were strictly fit on the training cohort and subsequently applied to the internal validation and external validation cohorts.

### Model construction and evaluation

Five machine learning (ML) methods, including support vector machine (SVM), logistic regression (LR), ridge regression, light gradient boosting machine (LightGBM), and extreme gradient boosting (XGBoost), were employed to construct clinical model (C), text model (T), radiomics model (R), text-radiomic fusion model (T + R), and text-radiomic-clinical fusion model (T + R + C) with selected features and clinical factors. The model demonstrating the best performance on the internal cohorts was selected for subsequent comparative analyses. Furthermore, a standalone assessment was performed on the external validation cohort by a senior radiologist with 10 years of experience to provide a clinical baseline. The radiologist, blinded to pathological results and model predictions, reviewed the original MRI reports and provided a binary DSI judgment for each case. The performance of this subjective assessment was then compared with the fusion models to evaluate their incremental clinical utility.

AUC of the receiver operating characteristic (ROC) curve, ACC, SEN, SPE, and precision (PRE) were calculated to evaluate the performance of models. The calibration of model-predicted DSI probabilities was evaluated using calibration curves and the Hosmer–Lemeshow (H–L) goodness-of-fit test. Delong test and decision curve analysis (DCA) were utilized to further evaluate the clinical utility of these models by quantifying the net benefits at different threshold probabilities in the training, internal validation, and external validation cohorts, respectively. The importance of individual features and their visualization to DSI were analyzed by using Shapley Additive exPlanation (SHAP).

### Statistical analysis

Statistical analysis was performed using the R analysis platform (version 4.3.0, http://www.Rproject.org). The used R packages are listed in Supplementary Table S3. For continuous clinical factors, data were expressed as means and standard deviations (SD) and compared using the Mann–Whitney *U*-test. Categorical factors were expressed as numbers and percentages and compared using the chi-square test. For all tests, *p* < 0.05 was considered statistically significant.

## Results

### Patients’ characteristics

Among 319 enrolled patients with pathologically confirmed ESCC, postoperative pathological analysis identified superficial stromal invasion in 117 (36.7%) and middle/deep stromal invasion in 202 (63.3%), respectively. The mean age of patients from hospital 1 (training: *n* = 210; internal validation: *n* = 89) and 2 (external validation: *n* = 20) was 56.67 ± 10.47, and 55.50 ± 10.08, respectively. Significant differences in age and tumor size were observed in the training cohort. Detailed clinical characteristics are listed in Table 1. Multivariate logistic analysis identified age (Odd ratio (OR) = 1.03, 95% confidence interval (CI): 1.00–1.06, *p* = 0.029) and tumor size (OR = 1.41, CI: 1.02–1.98, *p* = 0.039) as independent predictors of DSI risk for the C model, as shown in Table S4.

**Table 1:** Clinical characteristics of patients in the training, internal validation, and external validation cohorts

Characteristics	Training cohort			Internal validation cohort			External validation cohort		
	Superficial *n* = 75	Middle or deep *n* = 135	*p*	Superficial *n* = 32	Middle or deep *n* = 57	*p*	Superficial *n* = 10	Middle or deep *n* = 10	*p*
Age, years			0.020			0.018			0.161
Mean ± SD	54.44 ± 10.82	57.90 ± 9.67		53.19 ± 8.08	58.67 ± 12.12		51.90 ± 11.55	59.10 ± 7.22	
Size, cm			0.008			0.689			0.789
Mean ± SD	2.67 ± 1.00	2.96 ± 0.82		2.79 ± 0.96	2.85 ± 0.76		2.35 ± 0.73	2.60 ± 1.02	
LVSI			0.070			0.965			0.626
Positive	25	60		9	14		2	4	
Negative	49	65		21	38		8	6	
Unknow	1	10		2	5		0	0	
FIGO Stage			0.230			0.144			0.301
IA2	2	2		2	0		1	0	
IB1-2	49	74		19	33		6	4	
IIA1	24	59		11	24		3	6	
Tumor differentiation			0.411			0.531			1.00
Poorly differentiated	47	82		18	33		5	5	
Moderately differentiated	24	50		14	22		4	4	
Highly differentiated	4	3		0	2		1	1	

*p* value is calculated from the univariate association test between sub-groups

*χ*^2^ test and Fisher’s exact test for categorical variables; two-sample *t*-test for continuous variables

*SD* standard deviation, *LVSI* lymph-vascular space invasion, *FIGO* International Federation of Gynecology and Obstetrics

### Feature extraction and selection

**Fig. 2:**
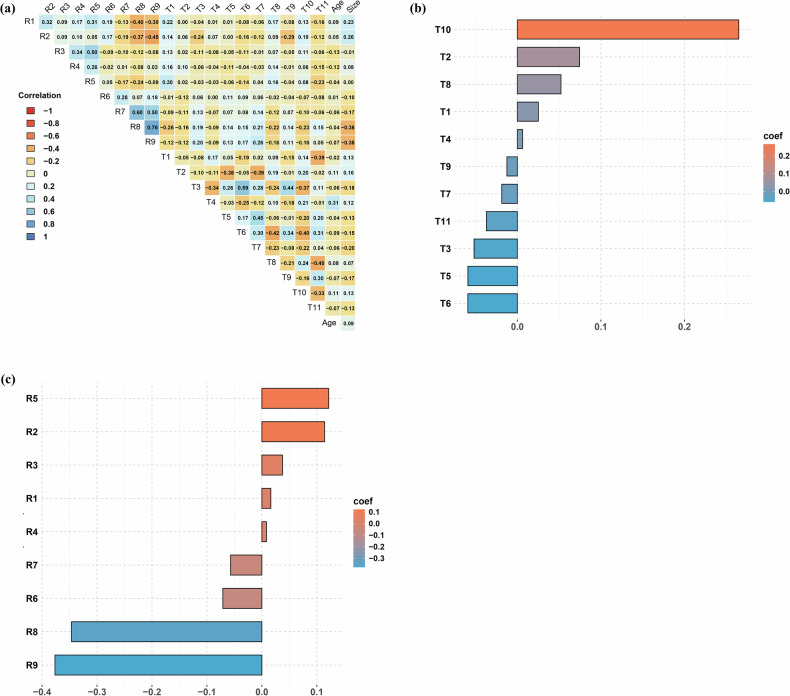
Spearman correlation analysis of all features (**a**), the coefficient weight of BERT features (**b**), and the coefficient weight of radiomic features (**c**)

A total of 498 radiomic features (from sagittal T2WI) and 384 BERT-derived features (from radiology reports) were extracted per patient. Following exclusion of features with poor reproducibility (ICC < 0.75), 314 reproducible radiomic features were retained for Spearman analysis. Subsequent feature selection via Mann–Whitney *U*-test and LASSO regression identified 11 BERT features and 9 radiomic features for modeling. The details of these key features are shown in Supplementary Table S5. All features showed inter-feature correlations < 0.7 (Fig. 2a). The BERT feature T10 and the radiomic feature R9 (GLZLM_SZE_2) exhibited the highest coefficient weights in their respective feature sets (Fig. 2b, c).

### Model evaluation

**Fig. 3:**
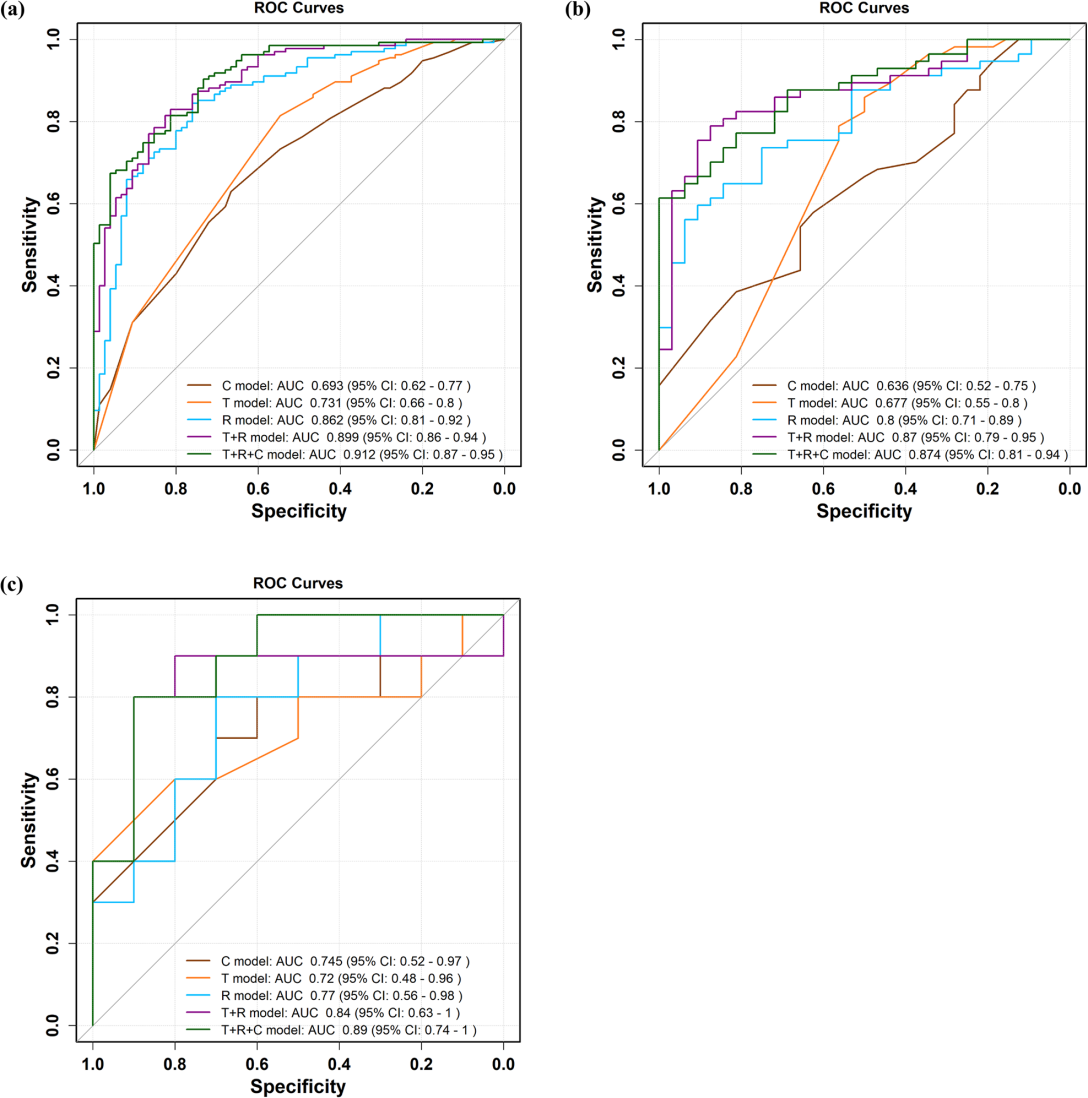
The AUCs of the C model, T model, R model, T + R model, and T + R + C model in the training (**a**), internal validation (**b**), and external validation (**c**) cohorts

Performance metrics (AUCs) for Models of C, T, R, T + R, T + R + C in the training, internal validation, and external validation cohorts were shown in Table S6. The results indicated that the LightGBM algorithm achieved the best performance among the five machine learning models in constructing both the C and T models, with AUCs of 0.693 (CI: 0.62–0.77) and 0.731 (CI: 0.66–0.80); 0.636 (CI: 0.52–0.75) and 0.677 (95% CI: 0.55–0.80); and 0.745 (CI: 0.52–0.97) and 0.720 (95% CI: 0.48–0.96) in the training, internal validation, and external validation cohorts, respectively (Fig. 3a–c and Table S6). Moreover, the XGBoost algorithm achieved the best performance for constructing both the R model and multimodal data fusion model (T + R and T + R + C). The AUCs of the R model and T + R were 0.862 (95% CI: 0.81–0.92) and 0.899 (95% CI: 0.86–0.94); 0.800 (95% CI: 0.71–0.89) and 0.870 (95% CI: 0.79–0.95); and 0.770 (95% CI: 0.56–0.98) and 0.840 (95% CI: 0.63–1.00) in the training, internal validation, and external validation cohorts, respectively (Fig. 3a–c and Table S6). The T + R + C demonstrated superior performance compared with the other models, with AUCs of 0.912 (CI: 0.87–0.95), 0.874 (95% CI: 0.81–0.94), and 0.890 (95% CI: 0.74–1.00) in the training, internal validation, and external validation cohorts, respectively (Fig. 3a–c and Table 2). The standalone radiologist assessment on the external validation cohort yielded an AUC of 0.650 (95% CI: 0.43–0.87), with an ACC of 0.65, SEN of 0.60, and SPE of 0.70 (Table 2).

**Table 2:** Performance of developed models across all cohorts and standalone radiologist assessment in the external validation cohort

Dataset	Models	AUC	95% CI	ACC	PRE	SPE	SEN	NPV	PPV
Training cohort	C model	0.693	0.62–0.77	0.64	0.77	0.67	0.63	0.50	0.77
	T model	0.731	0.66–0.80	0.72	0.76	0.55	0.81	0.62	0.76
	R model	0.862	0.81–0.92	0.81	0.86	0.76	0.84	0.73	0.86
	T + R	0.899	0.86–0.94	0.82	0.89	0.81	0.83	0.73	0.89
	T + R + C	0.912	0.87–0.95	0.84	0.86	0.73	0.90	0.81	0.86
Internal validation cohort	C model	0.636	0.52–0.75	0.60	0.73	0.63	0.58	0.45	0.73
	T model	0.677	0.55–0.80	0.73	0.75	0.50	0.86	0.67	0.75
	R model	0.800	0.71–0.89	0.71	0.92	0.91	0.60	0.56	0.92
	T + R	0.870	0.79–0.95	0.82	0.92	0.88	0.79	0.70	0.92
	T + R + C	0.874	0.81–0.94	0.75	1.00	1.00	0.61	0.59	1.00
External validation cohort	C model	0.745	0.52–0.97	0.70	0.67	0.60	0.80	0.75	0.67
	T model	0.720	0.48–0.96	0.70	0.75	0.80	0.60	0.67	0.75
	R model	0.770	0.56-0.98	0.75	0.73	0.70	0.80	0.78	0.73
	T + R	0.840	0.63–1.00	0.85	0.82	0.80	0.90	0.89	0.82
	T + R + C	0.890	0.74–1.00	0.85	0.89	0.90	0.80	0.82	0.89
	Standalone radiologist	0.650	0.43–0.87	0.65	0.67	0.70	0.60	0.64	0.67

*C* clinical, *T* text, *R* radiomic, *T* + *R* text-radiomic fusion, *T* + *R* + *C* text-radiomic-clinical fusion, *AUC* area under the curve, *CI* confidence interval, *ACC* accuracy, *PRE* precision, *SEN* sensitivity, *SPE* specificity, *NPV* negative predictive value, *PPV* positive predictive value

**Fig. 4:**
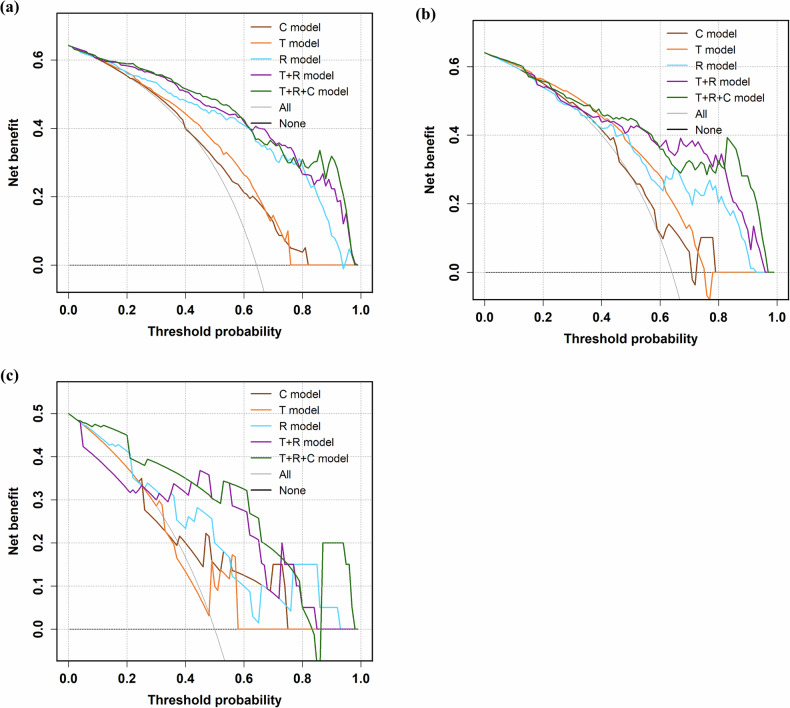
The DCA of the C model, T model, R model, T + R model, and T + R + C model in the training (**a**), internal validation (**b**), and external validation (**c**) cohorts

The calibration performance of the T + R + C model is summarized in Fig. S3. The calibration curves demonstrated excellent agreement between predicted probabilities and observed outcomes in all cohorts. This visual assessment was corroborated by H–L goodness-of-fit tests, which showed no significant deviation from perfect calibration in any cohort (training cohort: *p* = 0.200; internal validation cohort: *p* = 0.446; external validation cohort: *p* = 0.838). The DeLong test was applied to compare model AUCs and determine the statistical significance of their differences. In the training and internal validation cohorts, the T + R + C model demonstrated significantly better performance than both the C and T models (Fig. S4). However, the Delong test revealed no significant AUC differences between T + R and T + R + C models across all cohorts (training cohort: *p* = 0.497; internal validation cohort: *p* = 0.898; external validation cohort: *p* = 0.459). Although DeLong tests revealed no significant AUC differences between the T + R and T + R + C models across cohorts, DCA suggested that incorporating clinical variables may still provide incremental net benefit in decision-making (Fig. 4a–c).

### Model interpretation

**Fig. 5:**
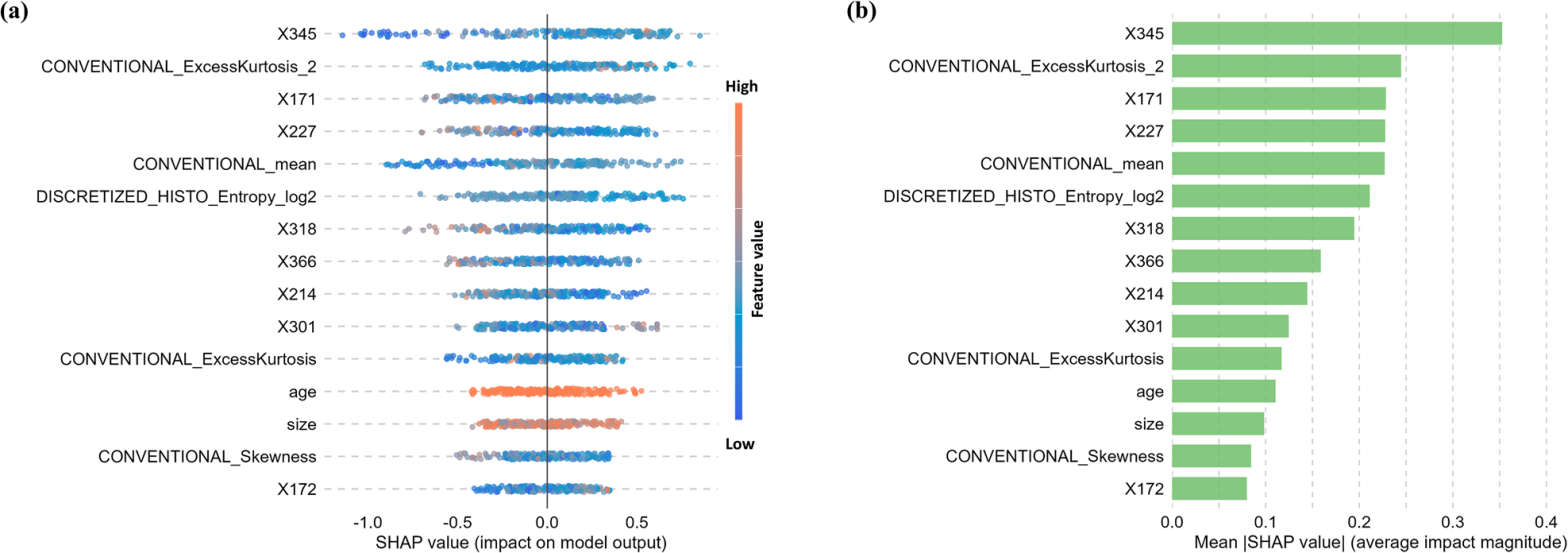
Feature importance based on SHAP values. Summary plot showing the impact of each feature on the model output, with color indicating the feature value (**a**). Mean SHAP value plot summarizing each feature’s average impact on prediction (**b**)

**Fig. 6:**
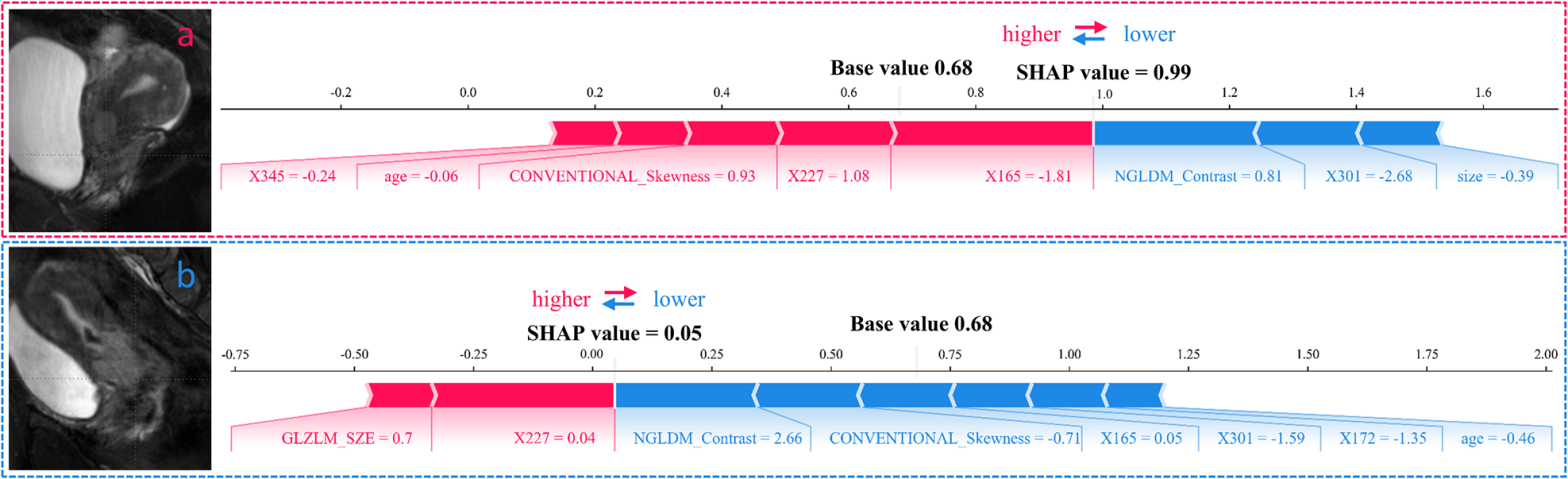
SHAP force plots illustrate how the T + R + C model discriminates between high-risk (**a**) and low-risk (**b**) DSI cases

SHAP analysis of the T + R + C model identified the top 15 predictive features for DSI risk (Fig. 5a, b), which comprised 8 BERT-based text features, 5 radiomic features, and 2 clinical features. This composition underscores the critical contribution of textual data to the model’s decision-making. Notably, the direction of each feature’s impact, as revealed by the SHAP summary plot (Fig. 5a), demonstrated clinical plausibility. For instance, higher values of key text features (e.g., X345) and specific radiomic features (e.g., CONVENTIONAL_ExcessKurtosis_2) were consistently associated with an increased predicted risk of DSI (positive SHAP values). This indicates that the model has learned to associate the complex semantic patterns encoded in the BERT features with radiological signs of deep invasion, providing a clinically meaningful interpretation of the text branch’s role in the multimodal framework. Furthermore, the force plot was utilized to interpret individual predictions. As shown in Fig. 6a, the SHAP value for the first patient was 0.99, which exceeded the base value of 0.68, indicating a high risk of DSI. Among the contributing features, the BERT-derived feature X165 (in red), with a value of −1.81, had a strong positive impact on the prediction of high DSI risk. In contrast, for another patient (Fig. 6b), the SHAP value was 0.05, which was lower than the base value, suggesting a low risk of DSI. In this case, the radiomics feature NGLDM_Contrast, with a value of 2.66, contributed negatively to the DSI risk assessment, reinforcing the low-risk prediction.

## Discussion

DSI serves as an independent prognostic indicator in ESCC and is significantly associated with tumor recurrence and increased mortality [31]. This study developed and validated a multimodal data fusion model that integrates clinical factors, radiomic features, and BERT-derived features from radiology reports to assess DSI in patients with ESCC. The multimodal data fusion model (T + R + C) demonstrated strong performance in predicting the risk of DSI, achieving AUCs of 0.912, 0.874, and 0.890 in the training, internal validation, and external validation cohorts, respectively.

Tumor size is a critical factor routinely assessed in conventional medical imaging analysis by radiologists to optimize the management of ESCC. A study has demonstrated that maximal tumor diameter (MTD) measured via MRI is an independent risk factor for DSI [32]. Similarly, our multivariate analysis identified age and tumor size as independent risk factors for the risk of DSI, as shown in Table S4. However, the clinical model based on these clinical factors achieved an AUC of 0.745 in the prediction of DSI, inferior to the reported AUC of 0.844 using MTD. This highlights the poor reproducibility of clinical factors for DSI prediction across studies.

Radiomic features extracted from MRI images have shown promise in DSI risk prediction for ESCC [14, 15]. A meta-analysis of 15 studies revealed that radiomics models achieved an overall SEN and SPE of 0.74 and 0.82 in the DSI risk prediction for endometrial cancer [33]. Our radiomics model similarly achieved an SEN and SPE of 0.80 and 0.70 in the external validation for DSI risk prediction. In this study, different ML algorithms were applied to optimize the DSI prediction with various features. The results demonstrated that the LightGBM algorithm outperformed other ML algorithms in C and T models, as shown in Table S6. Consistently, studies pointed out that LightGBM is able to reduce calculation time and allow missing values for prediction to achieve better generalization ability [34, 35].

NLP is widely applied to extract information from radiology reports for classifying disease occurrence, so as to aid clinicians in their decision-making and reduce workload [36]. Particularly, BERT models show promise for predicting oncological outcomes based on radiology reports by fine-tuning their pre-trained weights for specific tasks [37]. Promising applications in tumor response category classification and identification of genomic features associated with cancer outcomes have been reported [38, 39]. In this study, BERT-derived text features extracted from MIRs achieved AUCs of 0.731, 0.677, and 0.720 for DSI prediction in the training, internal validation, and external validation cohorts, respectively. Furthermore, a fusion model (T + R) improved the AUCs to 0.899, 0.870, and 0.840 in the training internal validation, and external validation cohorts, respectively, outperforming both models with radiomic or BERT features alone in the DSI risk prediction.

The superior net benefit of combined models of T + R and T + R + C was confirmed by DCA. SHAP values are widely recognized to enhance the interpretability and the impact of key features [40]. The top 15 most important features identified by SHAP analysis in the T + R + C model indicated that BERT features contribute significantly to the DSI prediction (8 out of 15 features). Radiomics features also contributed significantly to the combined model, with 5 first-order features (2 kurtosis features, 1 skewness feature, 1 mean feature, and 1 entropy feature) identified by SHAP. From a clinical perspective, accurate preoperative prediction of DSI has direct implications for surgical decision-making. Patients predicted to be at low risk may be considered for fertility-sparing approaches, whereas those at high risk may require radical hysterectomy and adjuvant therapy. Thus, the model’s predictions could serve as an adjunct to radiological and clinical assessment, supporting individualized treatment planning.

This study has several limitations. First, the retrospective design may introduce selection bias and limit causal inference, highlighting the need for future prospective validation. Second, deep learning (DL) has been applied to assess DSI risk in cervical cancer [41]. Prospective studies integrating DL with our methods with multicenter datasets are needed to further optimize prediction. Third, only squamous cell carcinoma cases were included, as this is the most prevalent subtype in CC. In addition, exclusion of tumors spanning fewer than three slices and variability in clinical characteristics within the training set may introduce spectrum bias, limiting generalizability to other histological subtypes, very small tumors, and broader patient populations. Fourth, while explicit DSI-related terms were removed, implicit radiological descriptors such as tumor morphology or signal heterogeneity correlated with DSI may still contribute to the text-derived signal. Consequently, the radiology-report model should be interpreted as an encoding of the radiologist’s clinical impression that effectively acts as a proxy for expert judgment, rather than as entirely independent biomarker information. Our benchmarking analysis further supports this, demonstrating that the multimodal model outperforms standalone radiologist assessments (AUC 0.890 vs 0.650) in the external validation cohort. Fifth, while the external validation cohort was relatively small, it consisted entirely of images from a different vendor (Philips vs GE) with distinct acquisition parameters. Although explicit feature harmonization (e.g., ComBat) was not applied, the model’s robust performance (AUC 0.890) on this completely unseen platform suggests that our rigorous feature selection and normalization pipeline partially mitigated scanner-dependent variability. Nonetheless, the absence of harmonization and nested cross-validation for model selection may still introduce optimism bias and affect radiomic reproducibility. Future large-scale, multi-center studies with standardized imaging protocols and advanced harmonization techniques are warranted to further confirm the model’s transportability and clinical utility.

## Conclusion

This study proposed a SHAP-based explainable multimodal fusion model integrating clinical, radiomic, and BERT-derived features, which demonstrated improved performance in preoperative DSI risk evaluation. While promising for individualized risk stratification, these preliminary findings necessitate further large-scale, multi-center validation due to the small external cohort to confirm generalizability before clinical implementation.

## Supplementary information

ELECTRONIC SUPPLEMENTARY MATERIAL

## Data Availability

The image data from the two centers cannot be shared publicly due to data privacy concerns and restricted permissions in the current study. However, anonymized data can be accessed under restricted conditions for patient privacy. Access requests for academic purposes can be made by contacting the corresponding author.

## Abbreviations

ACC: Accuracy
AUC: Area under the curve
BERT: Bidirectional encoder representations from transformers
DSI: Depth of stromal invasion
ESCC: Early-stage cervical cancer
FIGO: International Federation of Gynecology and Obstetrics classification
GLZLM: Gray-level zone length matrix
H–L: Hosmer–Lemeshow
ICC: Intraclass correlation coefficient
LASSO: Least absolute shrinkage and selection operator
LVSI: Lymph-vascular space invasion
MRI: Magnetic resonance imaging
NGLDM: Neighborhood gray-level different matrix
NLP: Natural language processing
PET/CT: Positron emission tomography/computed tomography
PRE: Precision
ROC: Receiver operating characteristic
SEN: Sensitivity
SHAP: Shapley additive explanation
SPE: Specificity
VOI: Volume of interest
