# Supplementary material for: MRI- and report-based multimodal model with SHAP-based explanation for preoperative prediction of deep stromal invasion in early-stage cervical cancer
Source: Insights Imaging. 2026 May 15;17:132. doi: 10.1186/s13244-026-02311-7 (PMC13179407; doi:10.1186/s13244-026-02311-7)
Supplement: Supplementary file 1 — ELECTRONIC SUPPLEMENTARY MATERIAL [file 13244_2026_2311_MOESM1_ESM.pdf]

# MRI- and Report-Based Multimodal Model with SHAP-Based Explanation for Preoperative Prediction of Deep Stromal Invasion in Early-Stage Cervical Cancer

## ELECTRONIC SUPPLEMENTARY MATERIAL


1. Figure S1: The study design and analytical approach of this research……………2
2. Figure S2: Representative image of the lesions for ESCC………..……………..….. 3
3. Figure S3: The calibration curve of the T+R+C in all cohorts……………………4
4. Figure S4: Delong test in all cohorts………………………………………………5
5. Table S1: The details of MRI scan protocol and imaging processing……………..... 6
6. Table S2: A detailed list of excluded keywords………………………………....7
7. Table S3: R packages……………..………………………………..……......…….8
8. Table S4: Multivariate logistic regression analysis for predictive factors of stromal invasion risk………..………………………………………………………..…9
9. Table S5: Key text and radiomic features selected by LASSO……………......…10

10. Table S6: Stromal invasion prediction performance of C, T, R, T+R and T+R+C models in the training, internal and external validation cohorts………………………………………………………………………………………….................... 11

**Figure S1**: The study design and analytical approach of this research

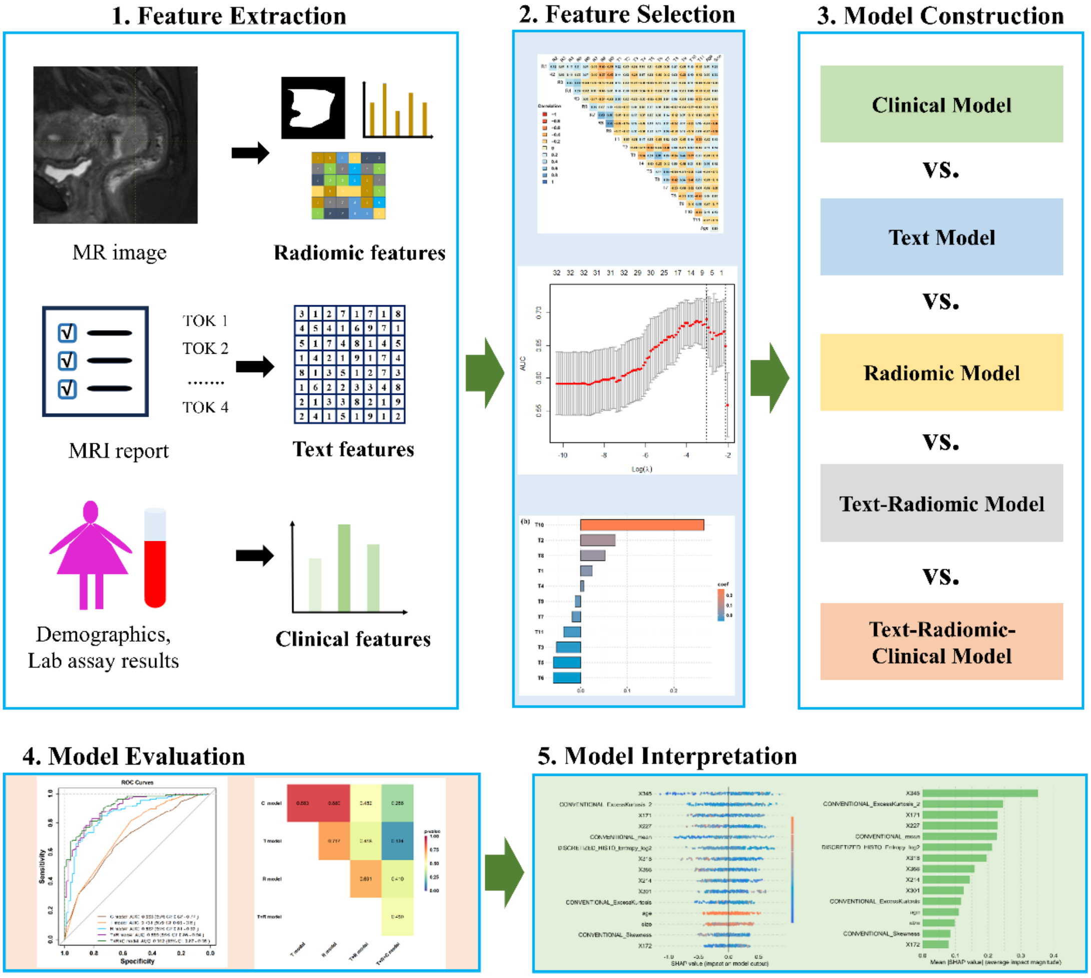

**Figure S2:** A representative image of the lesion for ESCC

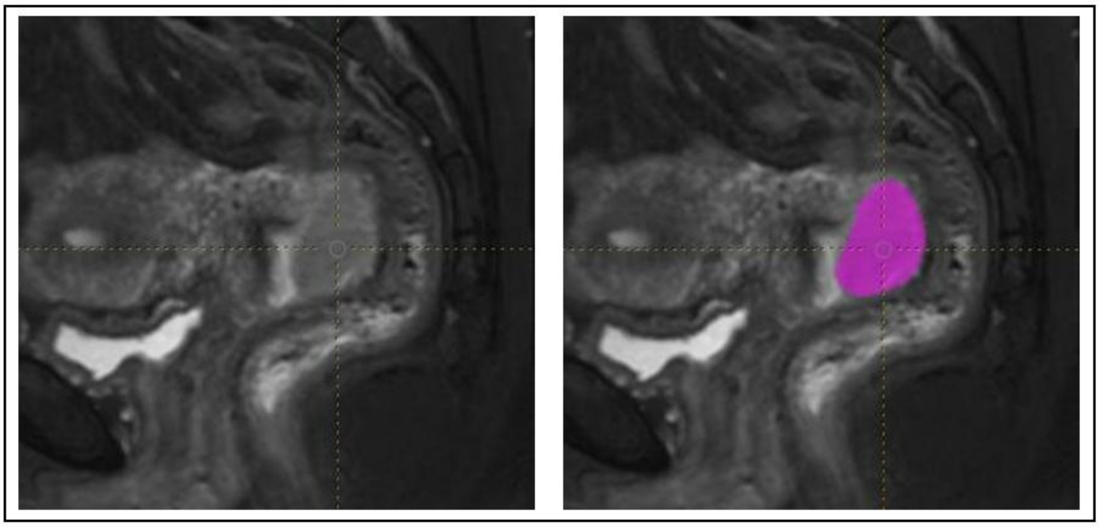

**Figure S3:** The calibration curve of the T+R+C in all cohorts

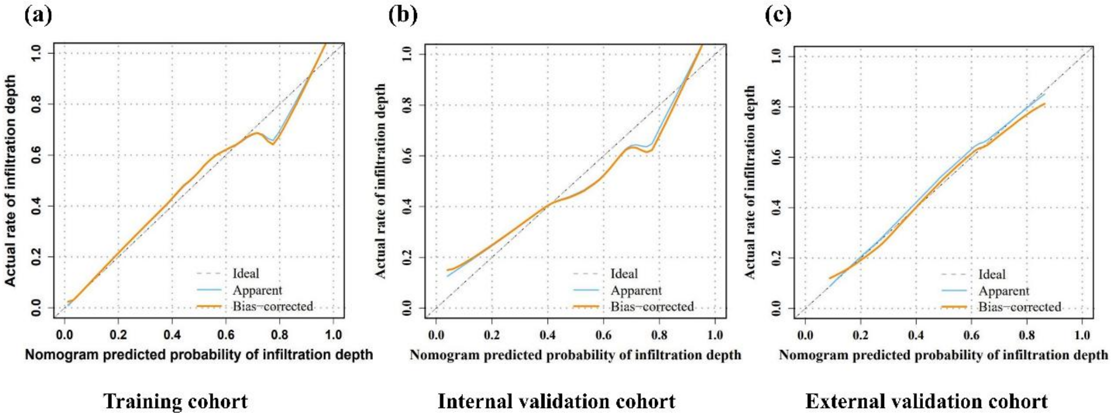

**Figure S4:** Delong test in all cohorts

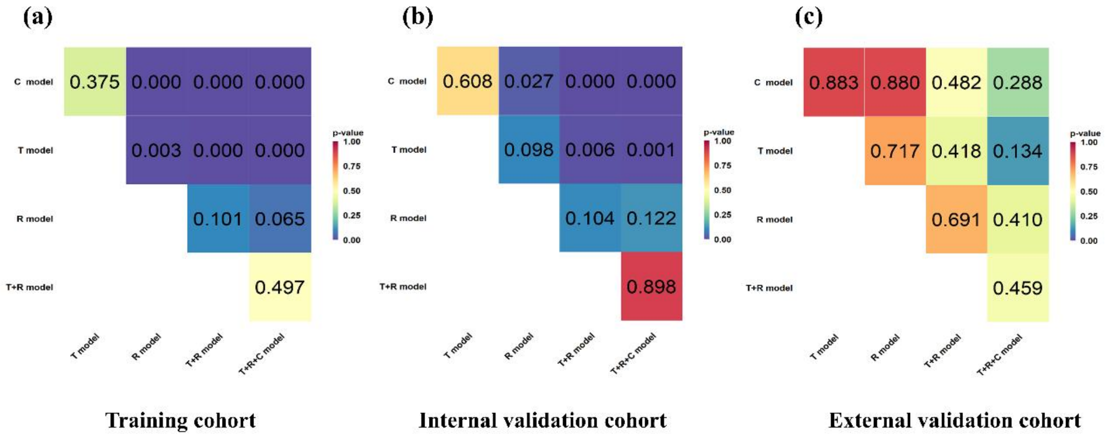

**Table S1:** The details of MRI scan protocol and imaging processing

| **Parameter** | Center 1 (GE Signa EXCITE/HDxT) | Center 2 (PHILIPS Achieva) |
|---|---|---|
| **Sequence Type** | Fast Spin Echo (FSE) | Fast Spin Echo (FSE) |
| **Field of View (FOV)** | 220×220 mm | 220×220 mm |
| **Matrix Size** | 320×320 | 336 × 336 |
| **Slice Thickness (mm)** | 4 | 4 |
| **Interslice Gap (mm)** | 1 | 1 |
| **Repetition Time (TR)** | 3500-4500 ms | 4000 ms |
| **Echo Tiem (TE)** | 90-110 ms | 100 ms |
| **Flip Angle** | 90° | 90° |

**Table S2** A detailed list of excluded keywords

| Category | Specific Chinese Terms | English Translation |
|---|---|---|
| Direct DSI Descriptors | 累及全肌层、累及全层、全层受累 | Full-thickness involvement |
| Direct DSI Descriptors | 纤维基质层中断、间质环中断 | Disruption of the fibrous stroma ring |
| Indirect Proxies (Stage ≥ II) | 宫旁受累、侵犯宫旁、阴道穹窿受累 | Parametrial involvement, Vaginal fornix involvement |
| Indirect Proxies (Invasion) | 突破浆膜、突破外膜、浆膜面不光整 | Serosal breakthrough, Irregular serosal surface |

**Table S3** R packages

| Numbers | Name |
|---|---|
| 1 | lpSolve |
| 2 | irr |
| 3 | pROC |
| 4 | e1071 |
| 5 | glmnet |
| 6 | ggplot2 |
| 7 | tidyverse |
| 8 | xgboost |
| 9 | skimr |
| 10 | DataExplorer |
| 11 | caret |
| 12 | Matrix |
| 13 | dplyr |
| 14 | rms |
| 15 | psych |
| 16 | regplot |
| 17 | readr |
| 18 | rmda |
| 19 | corrplot |
| 20 | ggcorrplot |
| 21 | vcd |
| 22 | ggrepel |
| 23 | ResourceSelection |
| 24 | caret |
| 25 | lightgbm |
| 26 | DMwR |
| 27 | Reshape2 |
| 28 | RColorBrewer |

**Table S4** Multivariate logistic regression analysis for predictive factors of stromal invasion risk

| Variables | Odds ratio | 95% CI | *P*-value |
|---|---|---|---|
| Age | 1.033 | 1.004 – 1.064 | **0.029** |
| Size | 1.413 | 1.023 – 1.977 | **0.039** |

OR: Odd ratio; CI: confidence interval.

**Table S5** Key text and radiomic features selected by LASSO

| Numbers | Radiomic features | Numbers | Text features |
|---|---|---|---|
| R1 | CONVENTIONAL_ExcessKurtosis | T1 | X165 |
| R2 | CONVENTIONAL_mean | T2 | X167 |
| R3 | CONVENTIONAL_ExcessKurtosis_2 | T3 | X171 |
| R4 | GLZLM_SZE | T4 | X172 |
| R5 | CONVENTIONAL_ExcessKurtosis_3 | T5 | X214 |
| R6 | CONVENTIONAL_Skewness | T6 | X226 |
| R7 | DISCRETIZED_HISTO_Entropy_log2 | T7 | X227 |
| R8 | NGLDM_Contrast | T8 | X301 |
| R9 | GLZLM_SZE_2 | T9 | X318 |
| | | T10 | X345 |
| | | T11 | X366 |

LASSO: least absolute shrinkage and selection operator; GLZLM: Gray-level zone length matrix; NGLDM: neighborhood gray-level different matrix; GLRLM: gray-level run length matrix

**Table S6** Stromal invasion prediction performance of C, T, R, T+R and T+R+C models in the training, internal and external validation cohorts

| Dataset | Cohorts | Models | AUC | 95% CI | ACC | PRE | SPE | SEN |
|---|---|---|---|---|---|---|---|---|
| C models | Training cohort | SVM | 0.627 | 0.55-0.71 | 0.66 | 0.73 | 0.51 | 0.74 |
| | | LR | 0.637 | 0.56-0.72 | 0.63 | 0.80 | 0.75 | 0.56 |
| | | Ridge | 0.637 | 0.56-0.72 | 0.63 | 0.80 | 0.75 | 0.56 |
| | | LightGBM | **0.693** | **0.62-0.77** | **0.64** | **0.77** | **0.67** | **0.63** |
| | | XGBoost | 0.690 | 0.62-0.76 | 0.68 | 0.76 | 0.57 | 0.73 |
| | Internal validation cohort | SVM | 0.639 | 0.52-0.75 | 0.63 | 0.79 | 0.72 | 0.58 |
| | | LR | 0.616 | 0.49-0.74 | 0.64 | 0.70 | 0.44 | 0.75 |
| | | Ridge | 0.616 | 0.49-0.74 | 0.64 | 0.70 | 0.44 | 0.75 |
| | | LightGBM | **0.636** | **0.52-0.75** | **0.60** | **0.73** | **0.63** | **0.58** |
| | | XGBoost | 0.678 | 0.56-0.80 | 0.64 | 0.79 | 0.72 | 0.60 |
| | External validation cohort | SVM | 0.800 | 0.59-1.00 | 0.80 | 0.88 | 0.90 | 0.70 |
| | | LR | 0.820 | 0.63-1.00 | 0.80 | 0.88 | 0.90 | 0.70 |
| | | Ridge | 0.820 | 0.63-1.00 | 0.80 | 0.88 | 0.90 | 0.70 |
| | | LightGBM | **0.745** | **0.52-0.97** | **0.70** | **0.67** | **0.60** | **0.80** |
| | | XGBoost | 0.650 | 0.40-0.90 | 0.65 | 0.64 | 0.60 | 0.70 |
| T models | Training cohort | SVM | 0.709 | 0.63-0.78 | 0.72 | 0.76 | 0.53 | 0.83 |
| | | LR | 0.719 | 0.65-0.79 | 0.71 | 0.78 | 0.61 | 0.77 |
| | | Ridge | 0.716 | 0.64-0.79 | 0.73 | 0.76 | 0.51 | 0.85 |
| | | LightGBM | **0.731** | **0.66-0.80** | **0.72** | **0.76** | **0.55** | **0.81** |
| | | XGBoost | 0.721 | 0.65-0.80 | 0.70 | 0.79 | 0.65 | 0.73 |
| | Internal validation cohort | SVM | 0.748 | 0.64-0.85 | 0.72 | 0.82 | 0.72 | 0.72 |
| | | LR | 0.737 | 0.63-0.84 | 0.67 | 0.85 | 0.81 | 0.60 |
| | | Ridge | 0.750 | 0.65-0.85 | 0.74 | 0.79 | 0.63 | 0.81 |
| | | LightGBM | **0.677** | **0.55-0.80** | **0.73** | **0.75** | **0.50** | **0.86** |
| | | XGBoost | 0.725 | 0.61-0.84 | 0.72 | 0.79 | 0.63 | 0.77 |
| | External validation cohort | SVM | 0.820 | 0.62-1.00 | 0.80 | 0.88 | 0.90 | 0.70 |
| | | LR | 0.860 | 0.68-1.00 | 0.85 | 1.00 | 1.00 | 0.70 |
| | | Ridge | 0.780 | 0.57-0.99 | 0.75 | 0.73 | 0.70 | 0.80 |
| | | LightGBM | **0.720** | **0.48-0.96** | **0.70** | **0.75** | **0.80** | **0.60** |

| | | | | | | | | |
|---|---|---|---|---|---|---|---|---|
| | | XGBoost | 0.710 | 0.46-0.96 | 0.75 | 0.78 | 0.80 | 0.78 |
| R models | Training cohort | SVM | 0.701 | 0.62-0.78 | 0.65 | 0.81 | 0.75 | 0.60 |
| | | LR | 0.745 | 0.68-0.81 | 0.75 | 0.77 | 0.53 | 0.87 |
| | | Ridge | 0.734 | 0.66-0.80 | 0.72 | 0.77 | 0.57 | 0.80 |
| | | LightGBM | 0.703 | 0.63-0.77 | 0.70 | 0.77 | 0.59 | 0.77 |
| | | XGBoost | **0.862** | **0.81-0.92** | **0.81** | **0.86** | **0.76** | **0.84** |
| | Internal validation cohort | SVM | 0.731 | 0.61-0.85 | 0.75 | 0.80 | 0.63 | 0.82 |
| | | LR | 0.681 | 0.57-0.80 | 0.58 | 0.88 | 0.91 | 0.40 |
| | | Ridge | 0.727 | 0.62-0.84 | 0.72 | 0.79 | 0.63 | 0.77 |
| | | LightGBM | 0.658 | 0.55-0.77 | 0.67 | 0.75 | 0.56 | 0.74 |
| | | XGBoost | **0.800** | **0.71-0.89** | **0.71** | **0.92** | **0.91** | **0.60** |
| | External validation cohort | SVM | 0.590 | 0.32-0.86 | 0.65 | 0.59 | 0.30 | 1.00 |
| | | LR | 0.520 | 0.24-0.80 | 0.65 | 0.64 | 0.60 | 0.70 |
| | | Ridge | 0.620 | 0.36-0.88 | 0.65 | 0.62 | 0.50 | 0.80 |
| | | LightGBM | 0.605 | 0.36-0.85 | 0.60 | 0.57 | 0.40 | 0.80 |
| | | XGBoost | **0.770** | **0.56-0.98** | **0.75** | **0.73** | **0.70** | **0.80** |
| T+R models | Training cohort | SVM | 0.768 | 0.70-0.83 | 0.71 | 0.83 | 0.73 | 0.70 |
| | | LR | 0.799 | 0.74-0.86 | 0.70 | 0.87 | 0.84 | 0.61 |
| | | Ridge | 0.784 | 0.72-0.85 | 0.69 | 0.88 | 0.85 | 0.60 |
| | | LightGBM | 0.757 | 0.69-0.82 | 0.74 | 0.82 | 0.71 | 0.76 |
| | | XGBoost | **0.899** | **0.86-0.94** | **0.82** | **0.89** | **0.81** | **0.83** |
| | Internal validation cohort | SVM | 0.803 | 0.71-0.90 | 0.76 | 0.89 | 0.84 | 0.72 |
| | | LR | 0.743 | 0.64-0.85 | 0.72 | 0.83 | 0.75 | 0.70 |
| | | Ridge | 0.794 | 0.70-0.89 | 0.76 | 0.86 | 0.78 | 0.75 |
| | | LightGBM | 0.742 | 0.64-0.85 | 0.72 | 0.85 | 0.78 | 0.68 |
| | | XGBoost | **0.870** | **0.79-0.95** | **0.82** | **0.92** | **0.88** | **0.79** |
| | External validation cohort | SVM | 0.720 | 0.49-0.95 | 0.70 | 0.83 | 0.90 | 0.50 |
| | | LR | 0.590 | 0.32-0.86 | 0.65 | 0.67 | 0.70 | 0.60 |
| | | Ridge | 0.740 | 0.51-0.97 | 0.70 | 0.64 | 0.50 | 0.90 |
| | | LightGBM | 0.570 | 0.30-0.83 | 0.60 | 0.58 | 0.50 | 0.70 |
| | | XGBoost | **0.840** | **0.63-1.00** | **0.85** | **0.82** | **0.80** | **0.90** |
| T+R+C models | Training cohort | SVM | 0.772 | 0.71-0.84 | 0.68 | 0.86 | 0.83 | 0.60 |
| | | LR | 0.808 | 0.75-0.87 | 0.75 | 0.85 | 0.77 | 0.73 |

| | | | | | | | |
|---|---|---|---|---|---|---|---|
| | Ridge | 0.786 | 0.72-0.85 | 0.72 | 0.83 | 0.75 | 0.71 |
| | LightGBM | 0.802 | 0.74-0.87 | 0.76 | 0.83 | 0.71 | 0.79 |
| | XGBoost | **0.912** | **0.87-0.95** | **0.84** | **0.86** | **0.73** | **0.90** |
| Internal validation cohort | SVM | 0.811 | 0.72-0.90 | 0.80 | 0.87 | 0.78 | 0.81 |
| | LR | 0.760 | 0.66-0.86 | 0.74 | 0.85 | 0.78 | 0.72 |
| | Ridge | 0.800 | 0.70-0.90 | 0.787 | 0.852 | 0.75 | 0.81 |
| | LightGBM | 0.757 | 0.66-0.86 | 0.69 | 0.85 | 0.81 | 0.61 |
| | XGBoost | **0.874** | **0.81-0.94** | **0.75** | **1.00** | **1.00** | **0.61** |
| External validation cohort | SVM | 0.720 | 0.49-0.95 | 0.70 | 0.83 | 0.90 | 0.50 |
| | LR | 0.620 | 0.35-0.89 | 0.65 | 0.71 | 0.80 | 0.50 |
| | Ridge | 0.750 | 0.52-0.98 | 0.75 | 0.78 | 0.80 | 0.70 |
| | LightGBM | 0.530 | 0.26-0.80 | 0.60 | 0.56 | 0.30 | 0.90 |
| | XGBoost | **0.890** | **0.74-1.00** | **0.85** | **0.89** | **0.90** | **0.80** |

C: clinical; T:text; R:radiomic; T+R: text-radiomic fusion; T+R+C: text-radiomic-clinical fusion; SVM: support vector machine; LR: logistic regression; Light GBM: light gradient boosting machine; XGBoost: extreme gradient boosting; AUC: area under the curve; CI: confidence interval; ACC: Accuracy; PRE: Precision; SPE: Specificity; SEN: Sensitivity.
